# The effect of the Ontario stay-at-home order on Covid-19 third wave infections including vaccination considerations: An interrupted time series analysis

**DOI:** 10.1371/journal.pone.0265549

**Published:** 2022-04-06

**Authors:** Fatemeh Navazi, Yufei Yuan, Norm Archer

**Affiliations:** DeGroote School of Business, McMaster University, Hamilton, Ontario, Canada; University of South Carolina College of Pharmacy, UNITED STATES

## Abstract

The Covid-19 global pandemic that began in March 2020 was not fully mitigated through governmental Non-Pharmaceutical Interventions (NPIs) and continued to infect people and take lives through 2021. Since many countries were affected by the second, third, and fourth waves of Covid-19, governments extended and strengthened NPIs, but these actions led to citizen protests and fatigue. In this study, we investigate the effect of a lockdown policy on Covid-19 third wave implemented by the province of Ontario, Canada, on April 3^rd^ 2021, followed by a stay-at-home order on April 7^th^ 2021 while free Covid-19 testing and vaccination were in progress. Herein, the effect of both NPIs and vaccination are considered simultaneously. We used the prevalence of Covid-19 cases, tests, and administered vaccines data reported publicly by the Government of Ontario on their website. Because mobility changes can reflect the behaviors and adherence of residents with a stay-at-home order, Covid-19 community mobility data for Ontario provided by Google was also considered. A statistical method called interrupted time series was used to analyze the data. The results indicated that, although vaccinations helped to control the Covid-19 infection rate during this time, the stay-at-home order caused a rate reduction by decreasing the trend of the Covid-19 prevalence by 13 (±0.8962) persons per million daily and the level by 33 (±7.6854) persons per million. Furthermore, the stay-at-home order resulted in approximately a 37% reduction in Covid-19 prevalence one week after the intervention’s effective date. Therefore, Ontario’s strict lockdown policy, including several NPIs, mitigated the Covid-19 surge during the third wave. The results show that even when vaccination is in progress, strict NPIs such as lockdown is required to control Covid-19 waves, and early re-openings should be avoided. These results may also be useful for other countries that have implemented delayed vaccination schedules.

## Introduction

Due to the widespread viral outbreak of Covid-19 at the end of 2019, the World Health Organization (WHO) declared it a global pandemic on 11 March 2020 [1]. In the early Covid-19 era, in which no effective medications and vaccines were available, many governments employed various Non-Pharmaceutical Interventions (NPIs) to control the spread of Covid-19 infections [2]. Various researchers have evaluated the effectiveness of different types of NPIs: restrictive NPIs and supportive NPIs. Restrictive NPIs try to limit resident contacts to control individual to individual Covid-19 exposure, and supportive NPIs try to support residents and businesses by providing free Covid-19 testing, public surface cleaning and disinfecting, financial help for individuals or small businesses that lost their jobs, etc. It is important to study the effectiveness of NPIs because NPIs successfully reduced the Covid-19 death rate in several countries [3]. Brauner et al. compared the effectiveness of NPIs in a cross-country study using the Bayesian inference method [3]. They inferred that while the effect of school and business closures and limiting gatherings to control Covid-19 were considerable, the additional stay-at-home order was less effective. Investigating the effectiveness of NPIs is essential because they can also cause adverse socioeconomic issues, and their use must be balanced against the ability to mitigate the spread of the targeted infection [4].

By reviewing the literature, previous studies can be divided into two groups: studies that investigated the effectiveness of several NPIs to help choose the most effective one (i.e., according to Askitas et al. [4], canceling public/private gatherings and school and workplace closure are the most effective NPIs; whereas stay-at-home requirement and workplace /school closures are the most effective NPIs, found by to Wibbens et al. [5]), and studies that investigated the effect of a single NPI on different criteria such as Covid-19 reproduction rate, growth rate, incidence rate/cases [6], deaths [6], and cumulative rates [7]. The problem with these single policy studies is that they could not separate the results of other ongoing preventive measures from specific NPI results.

However, some comprehensive NPIs consist of several Covid-19 control policies. Results obtained by studying these NPIs can truly represent how they affect the spread of Covid-19. “Lockdown” is one of these control policies, consisting of several restrictive policies. Lockdown is a package of policies such as non-essential business closure [8], mandatory face mask use in businesses that provide essential services, social and physical distancing, public event and mass gathering bans, restrictions on the number of people in gatherings, school closures replaced by online and remote education, restrictions on international and internal travel, including border closures, temperature screening, and testing of travelers who are permitted to travel only for essential reasons, according to Ontario grey lockdown zone rules [9]. Kwak et al. used a deep reinforcement learning algorithm- an extension of the machine learning method- to find the optimal level of lockdown, which helps to decelerate Covid-19 incidence and death [10]. After running the algorithm, the agent (intelligent decision-making) suggested earlier implementation of lockdown even before the first wave began.

There is a slight difference between a lockdown and stay-at-home order. A “stay-at-home order” policy is implemented when lockdown measures are accompanied by workplace closures and more restrictive levels of other policies like limitations on the number of people/employees who are allowed to gather in a place [11]. In a stay-at-home order, private sector companies are required to limit their workplace capacity and enable their employees to work from home as much as possible [12]. Public health authorities and policymakers may also ask residents to decrease their mobility and not travel beyond their residential areas unless essential [13]. Unfortunately, these restrictive measures come with economic burdens.

Cowling et al. studied interventions in the early Covid-19 era in Hong Kong and showed that strict policies like lockdowns could be replaced by social distancing and behavioral changes [14]. In addition, Bendavid et al. compared some countries with South Korea and Sweden, which did not implement mandatory stay-at-home orders and business closures. They concluded that strict policies could be replaced effectively by less restrictive policies [8]. However, choosing countries like South Korea with specific characteristics (a peninsula with few borders) as the control group makes this comparison unfair. Furthermore, in a study by Brauner et al., the stay-at-home order affected the reproduction rate of Covid-19 by 15% more than a collection of several NPIs during the first wave [3]. In addition, Fowler et al. showed that stay-at-home order caused a reduction in Covid-19 incidence in states that implemented this policy during the first wave [15]. However, these studies were all related to the Covid-19 first wave, when effective vaccines were unavailable. A knowledge gap exists on the effect of the stay-at-home order while vaccination is in progress because of pharmaceutical intervention (vaccination) and non-pharmaceutical interventions’ interaction.

Most researchers in Covid-19 literature studied the first wave of Covid-19, but the data they used had some limitations at that time. For example, data from the early Covid-19 era displayed many uncertainties because of the shortage of Covid-19 test kits and the inaccuracy of some testing methods that could result in underestimation [16]. Also, no Covid-19 vaccine was available during the first wave, but several were available during the third wave, so the impact of vaccines must be considered when studying the third wave.

This study evaluated the effectiveness of a stay-at-home order when the population is partially vaccinated. Ontario, Canada was selected as a suitable case to study because the ultimate level of the stay-at-home order was implemented uniformly over the entire province.

### Previous history of Covid-19 in Ontario, Canada

Despite stringent Covid-19 preventive measures in 2020–2021, 545,000 Covid -19 cases were diagnosed in Ontario up to June 10^th^ 2021 [17]. The daily number of new Covid-19 cases in Ontario from the first case until June 10^th^ 2021, one day before re-opening, is illustrated in the S1 Fig [18]. During that time, Covid-19 caused 8902 deaths [17].

During the second Covid-19 wave the “Keeping Ontario Safe and Open” framework was implemented, with different Ontario regions classified into different color codes based on severity levels of infection [9]. Public health measures were adjusted and changed based on these color codes [9]. After exiting the second Covid-19 wave, the Premier of Ontario announced that on February 16^th^, 2021, lockdown would terminate in most of the 34 health regions, and they would exit from the grey lockdown zone [19], with businesses could re-opening in some regions (except the heavy populated Toronto, Peel, York regions). On March 15^th^ 2021, the government decreased the severity of control policies by adjusting capacity limits for some events in the grey zones [20].

However, when Ontario entered the third wave, policymakers decided to apply the stay-at-home order to all the health regions in Ontario. Because regional policy-making allowed people from grey cities like Toronto to travel to small cities with better conditions like Niagara Falls for leisure or to shop in Mississauga, the effectiveness of regional control policy-making failed in some periods; therefore, they paused the color code-based “Keeping Ontario Safe and Open” Covid-19 response framework. By announcing an “emergency brake” on the first of April, a stricter level of coordinated lockdown policy called “shut down” was implemented, starting from April 3^rd^ 2021 [21]. Also, the government asked residents to stay at home and leave their homes only for emergency needs through a stay-at-home order on April 7^th^ 2021 [11]. This was the second time since the beginning of the Covid-19 pandemic that Ontario had imposed a stay-at-home order. Furthermore, Ontario strengthened the enforcement of the stay-at-home order on April 17^th^ 2021 and even more on April 19^th^ 2021 by limiting inter-provincial travel to the neighboring provinces of Manitoba and Quebec [22]. This stay-at-home order was extended for longer periods on May 20^th^ 2021 and on June 2^nd^ 2021 before the provincial re-opening on June 11^th^ 2021 [23].

As observable in S1 Fig, the time distance between the second and third waves of Covid-19 was very short, and Ontario faced the third wave quickly. So, the performance of Covid-19 NPIs and the duration of lockdown is still under question to avoid swift occurrence of the fourth wave. As both the impacts of vaccinations and NPIs are of interest, and the third wave includes a significant number of vaccinated people and there was more testing available, the stay-at-home order during the third wave was chosen to be studied. In addition, during the third wave, the government implemented NPIs (including the stay-at-home order) uniformly to the entire province and not piecemeal among different Ontario regions as they did during the second wave. The research question for this study is: What would have happened during the third wave if there were no such strict restrictions?

## Materials and methods

In this section, we will assess the following hypotheses:

1. Was the “lockdown” Covid-19 preventive measure on April 3^rd^, emphasized by a stay-at-home order on April 7^th^ necessary and effective? Did this cause an initial level change (jump/fall) or a sustainable decreasing trend in the Covid-19 prevalence? What would have happened if just the lockdown (similar intervention with lower severity level) was continued?
2. Did the cumulative percentage of one-dose vaccinated people affect the Covid-19 prevalence? Because of shortages of the supplied vaccines, the Ontario government vaccination policy was to delay the second dose of vaccine to increase first dose administration.

### Data sources

The daily number of new Covid-19 cases regularly gathered from public health units across the province was extracted from the Ontario government website [18]. We utilized the cumulative daily number of people who had their first dose reported by the Ontario government on their website [24]. The daily number of Covid-19 tests in Ontario was obtained from the Ontario government website [25], based on the Ontario government’s daily epidemiological summary [26]. The cumulative percentage of one dose vaccinated people, Covid-19 prevalence, and population test rates were calculated by dividing these numbers by Ontario’s estimated 2021 population.

The center of excellence for the Canadian federations working with the Oxford Covid-19 government response tracker team [27] provides regional data for different Covid-19 control policies. We used the Ontario section of their data to verify the dates that the Ontario government’s control policy levels were changed from the provided regional Oxford stringency index.

Measuring the stringency of a particular policy is not enough. Another important facet is compliance with Covid-19 control policies, which can be measured by citizen mobility change patterns over time (if the nature of the policy targets mobility) [28]. Mobility is the frequency that people move around in a particular area. Companies like Google can obtain an estimate of area mobility based on the location information of people who enable location sharing in their Google accounts. Our study used 2021 mobility change data provided by Google LLC for Ontario residents which has no missing data for the studied period (March to May 2021) [29]. The mobility changes are calculated by comparing current mobility with the baseline, the mobility median value for the corresponding day of the week, during the pre- Covid-19 era of Jan 3^rd^ to Feb 6^th^, 2020 [30]. For example, on Thursday the first of April, the mobility change is 13%, which means Ontarians spent 13% more time at their home at that time. Google reports mobility changes in 6 areas, including retail and recreation, grocery and pharmacy, transit stations, workplaces, residential areas, and parks. Mobility in residential areas is the duration that people spend at their places of residence, while mobility in non-residential areas is the total number of visits at these places. Canada’s 2021 mobility change data are available in the regional CSV file of the Google mobility web page [29]. While two sub-region levels of data are provided, the data at the sub-region 1 (province) level was utilized. Google’s Covid-19 community mobility report was applied according to its terms and conditions to use in “understanding the data” section. As such, weekends and weekdays are separated with different colors in related Figs, and residential and non-residential mobilities are defined differently. Based on these mobility data, compliance with different policies that target the community, such as a stay-at-home order, can be specified.

Although the aforementioned data is publicly available in the cited references, their aggregated version is available for ease-of-use at http://dx.doi.org/10.17632/7k7rzy6t9g.1.

### Study design

As the whole Ontario population was subject to the NPIs imposed by the Ontario government, this is quasi-experimental research. According to Statistics Canada, the population of Ontario in 2020 was 14,734,014 with estimated population density of 13.688 persons/km^2^ [31].

To analyze the effect of the NPIs on the COVID–19 prevalence, a time interval needed to be selected. But there is no clear definition of an epidemic wave in the literature [32], so Zhang et al. [32] attempted to define an epidemic wave. They proposed that a wave is signaled when there is a rise in the number of new cases in such a way that at least for 14 days, the reproduction rate (*R*_*t*_) is greater than 1. The reproduction number of Covid-19 in Ontario provided by the Government of Ontario, (2021a) [18] confirms that March 7^th^ 2021 was the start of the third wave because, starting at March 7^th^ 2021, the reproduction rate became greater than one for more than 14 consecutive days. Zhang et al. also stated that when a containment strategy is successful, new infections decrease for a sustained period [32]. The start of the third wave was March 7^th^, 2021, when the level of new Covid-19 cases rose to 1300 (~1299). The peak of the third wave was April 16^th^, 2021, when there were 4812 new Covid-19 cases. The end of the third wave was May 31^st^, 2021, the date when less than 1000 new cases were reported.

Our study analyzed the effect of the severe lockdown policy (shut down) and the stay-at-home order on the third Covid-19 wave across Ontario. The shut down was province-wide and applicable to all 34 public health units of Ontario, similar to the strictest tier in the province’s Covid-19 control framework. This prohibits restaurant dining-in, personal care services, gym and outdoor classes, in-person shopping, and mass gatherings over five people. Schools were closed (except elementary and childcare centers), work from home was required (except where the nature of work made this impossible, and then only with limited capacity), international, intra-, and inter-provincial travel was allowed only for essential reasons, and face-coverings were mandatory in indoor areas or organized events like vaccination centers.

As part of the study design other variables were considered. First, the Ontario vaccination policy was to vaccinate as many people as possible with the first dose, so the percentage of people who received their first dose of the Covid-19 vaccine was considered. Besides the time series specifications, the number of Covid-19 tests representing the healthcare system’s capacity was considered, in addition to mobility changes in the population, including their behavior regarding stricter intervention.

### Descriptive data analysis

Based on Covid-19 control policy announcement dates, the stay-at-home order was announced on April 7^th^ 2021 [33]. Moreover, from the regional Oxford Stringency Index (OSI) (Composition of 9 Covid-19 mitigation policies, ranging from 0 to 100 (100 = strictest)) [34], the stringency index was sustained at levels of roughly 53 starting February 10^th^ 2021, increased to 62.1 on April 3^rd^ 2021, and then increased to 65.2 on April 8^th^ 2021 (including a one day delay from the day that the stay-at-home order was announced), and finally increased to 83.3 on April 19^th^ 2021 (based on the April 16^th^ government of Ontario announcement that some preventive measures were in place since April 17^th^ and more since April 19^th^). This confirms the stay-at-home order policy implementation date of April 7^th^, 2021, as stated in Ontario news [33]. So, a variable 0 before the stay-at-home order (April 7^th^, 2021) and 1 after that, was determined to measure level change. Moreover, a variable for trend change was added, defined as the number of days since implementing the new Covid-19 control policy.

**Fig 1:**
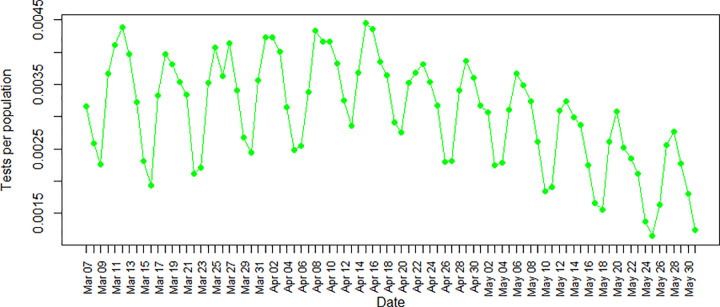
Number of tests per population during the third wave of Covid-19 in Ontario.

No outliers were detected in the data used in this study. Fig 1 illustrates the percentage of tests over time. As seen in Fig 1, the percentage of tests decreased on the weekends. Policymakers should find a solution for weekends to reduce the probability of patients with positive Covid-19 who are unaware of their infections and can infect people they might contact by not being in self-quarantine.

**Fig 2:**
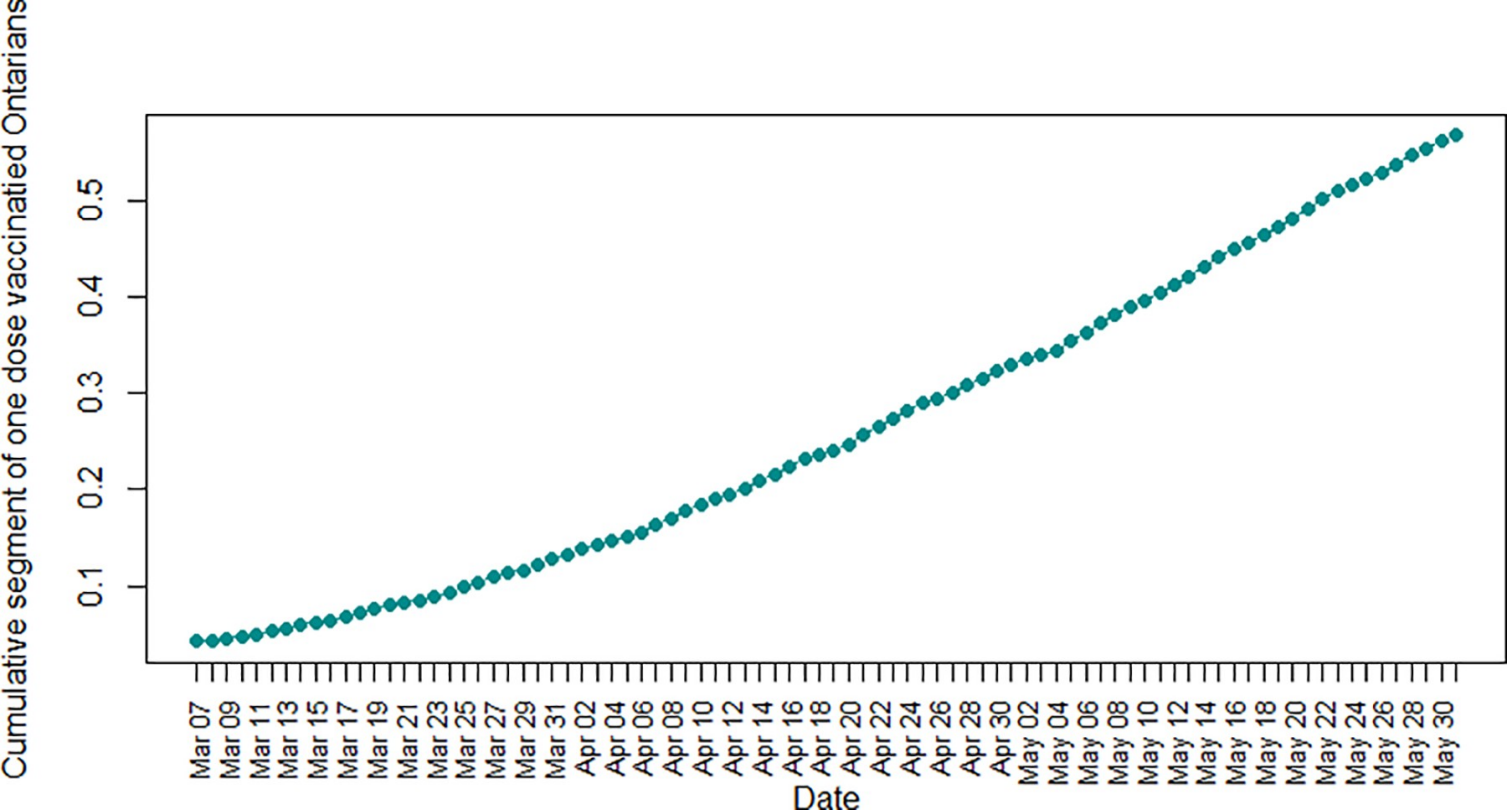
Cumulative segment of the first dose vaccinated Ontarians during the third wave of Covid-19.

Fig 2 illustrates the cumulative segment of Ontarians who got their first dose of the Covid-19 vaccine, which increased smoothly from less than 5 percent on March 7^th^, 2021, to more than 55 percent on May 31^st^, 2021.

**Fig 3:**
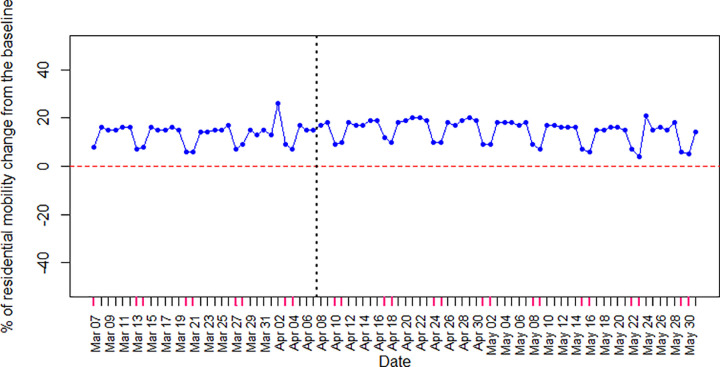
The percentage of change in residential mobility from the baseline in Ontario during the Covid-19 third wave.

We divided the mobility changes reported by Google LLC into two categories: residential and non-residential mobility changes. The residential mobility change shows the length of time people stayed at home. Fig 3 shows a positive percentage of residential mobility change from the baseline (around 15%) which illustrates residents’ permanence and compliance with Covid-19 control measures. As seen in Fig 3, residential mobility increased slightly for at least one month after the stay-at-home order announcement in April (vertical dotted line). Then, residential mobility decreased in May, displaying general frustration with the order. Considering only weekdays, the two samples *t*-test by Minitab 17 software showed that the means of residential mobility changes were not equal, and the difference before and after the stay-at-home order had (-2.975, -0.597) 95% confidence interval with *p*-value ≤ 0.01 (*p*-value = 0.004). In addition, the *p*-value of the mean difference one month before and after the stay-at-home order is more significant (*p*-value ≤ 0.001 (*p*-value = 0). Fig 3 also demonstrates weekend residential mobility change decreases, showing that people adhere less to the orders on weekends. Saturdays and Sundays are indicated in pink on the X-axis.

**Fig 4:**
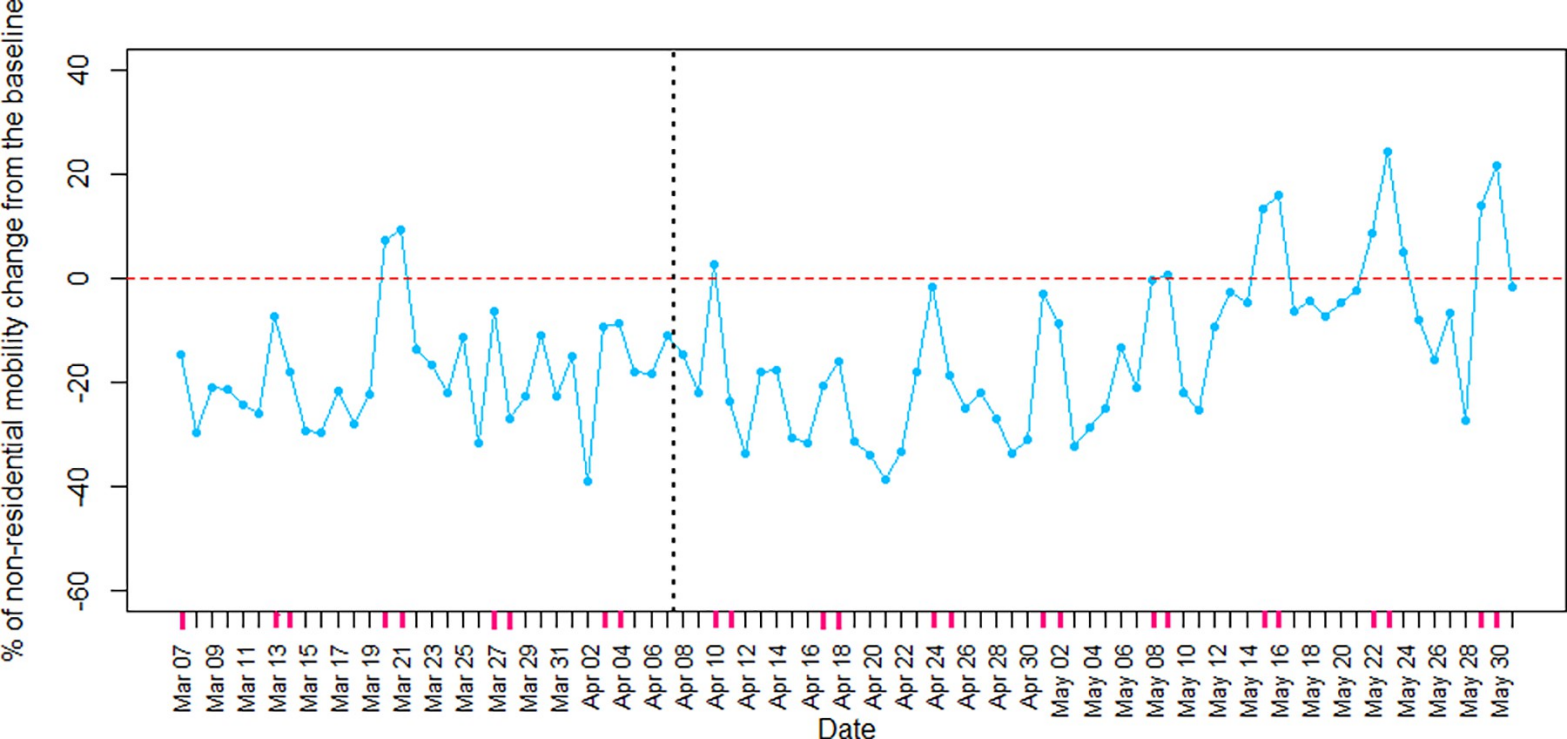
The percentage of change in non-residential mobility from the baseline in Ontario during the Covid-19 third wave.

In contrast, an increase in mobility of non-residential areas shows noncompliance with the stay-at-home order. The average percentage of non-residential mobility change in Fig 4 shows the fluctuation in the number of visitors of five non-residential areas, including retail and recreation areas, grocery and pharmacy, transit stations, workplaces, and parks. The negative percentage indicates that people spent less time in non-residential areas than usual. Fig 4 also shows a decrease in non-residential mobility change for at least 3 weeks after the stay-at-home order announcement. Also, considering only weekdays, the two samples *t*-test showed a significant 5.22 mean decrease (from -22.05 to -27.27) in non-residential mobility change three weeks after the intervention (until the end of April) with (0.53, 9.91) confidence interval at 95% with *p*-value ≤ 0.05. Although people seemed to adhere to the second stay-at-home order in April, the mobility in non-residential areas increased in May. Also, the two samples *t*-test showed significant 14.67 units increase in non-residential mobility change in May compared to April (after intervention) with (8.71, 20.63) confidence interval at 95% level and *p*-value ≤ 0.05. This tends to be related to the mobility increase in public parks because of good weather or people’s exhaustion from staying at home for a long time. Google also reported a correlation between weather temperature and mobility increase in public parks.

Furthermore, Hu et al. used a big data analytics tool and showed that policies have a limited and time decreasing effect on the USA population mobility [35]. The following section discusses the main analysis of the data.

### Statistical analysis by Interrupted Time Series (ITS)

A variety of research methodologies, including case-control studies, time series, interrupted time series, case studies, and epidemy mathematical modeling, have been utilized to evaluate the effectiveness of Covid-19 prevention policies [36]. When governmental interventions are implemented at a specific time, they can cause an interruption in the trend of ongoing Covid-19 time series (e.g., new Covid-19 case time series, number of deaths caused by Covid-19 time series, etc.). Therefore, interrupted time series is a suitable statistical method to analyze the effect of governmental interventions. If changing the governmental policy caused an interruption in the pattern, the level and slope of time series and intervention parameters are statistically significant.

**Fig 5:**
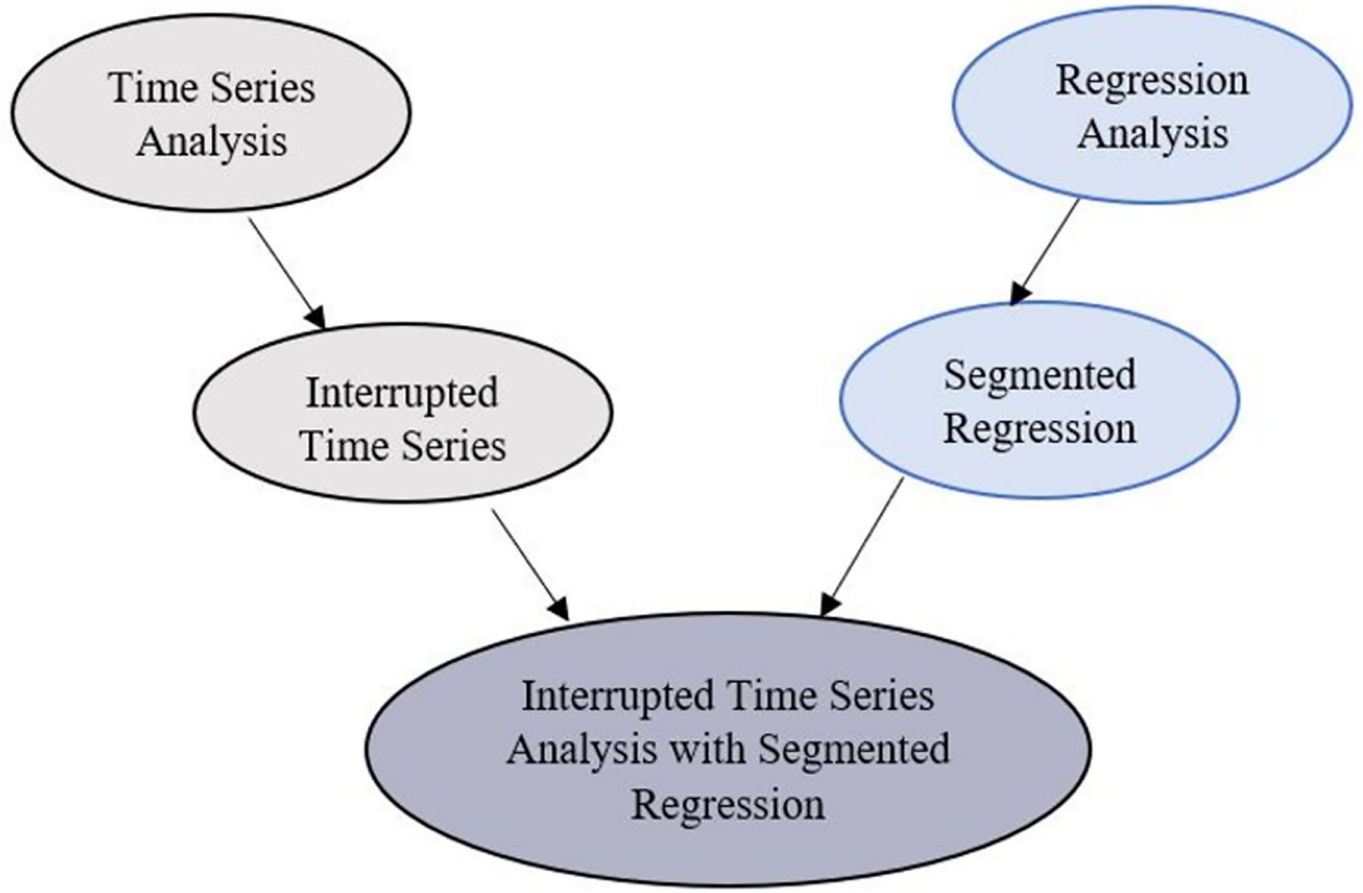
Placing utilized method among a hierarchy of related methods.

Interrupted Time Series (ITS) can be categorized as one of the regression discontinuity design subcategories. Regression discontinuity design is a pretest-posttest design that tracks the intervention’s causal effect by assigning a cut-off above or below the intervention. ITS is a well-known method for evaluating public health interventions [36], so it can help policy/decision-makers to see if their policy is effective or not. Two components related to the intervention, which differs before and after implementing that intervention, including level change and trend change, should be considered in a regression line besides time series variables to assess the intervention’s effectiveness [37]. This way, an interrupted time series is analyzed by the segmented regression method that assigns different regression lines to different segments [38]. Fig 5 locates the utilized method among its upstream methods.

Several papers that used interrupted time series for analyzing NPI effectiveness are as follows:

Auger et al. used interrupted time series to analyze the effect of school closure intervention timing while considering some Covid-19 control policies as covariates in a stepwise regression model in some US states [16]. However, their limitation was that the reported reduction could be related to other NPIs, which were also in place at that time. Sensitivity analysis around the lag time of school closure indicates that the states that employed closure earlier experienced a higher reduction of Covid-19 incidence and mortality. In emergency management, a plan’s timing is crucial to rescue more people [39]. Auger et al. studied the effectiveness of a single school closure policy [16]. Similarly, Emeto et al. studied border closure (a single policy) impact on Covid-19 incidence rate via an interrupted time series analysis [40]. They assumed a 7-day lag for implementing this policy in 9 African countries. However, they did not consider the incubation period of Covid-19. This may be why their results showed minimal effect of border closure on Covid-19 incidence rate. They mentioned that the enhanced testing and surveillance activities might have influenced their analysis. Hence, the number of Covid-19 tests per day was included in our interrupted time-series study to offset this factor.

Furthermore, Saki et al. used an interrupted time series to analyze the impact of social distancing (a single policy) on Covid-19 incidence cases and deaths in Iran [6]. They also used Ordinary Least Squares (OLS) regression to estimate their time series model coefficients. It is apparent from their results that they applied a linear model, as both the projected trend and observed trend were linear. However, Covid-19 prevalence usually has a non-linear relationship with time that can be captured by adding a quadratic term, which we applied in this study. Results from Saki et al. indicated the effect of social distancing policy in decreasing the total number of deaths and new cases that may have depended on other policies implemented by the government.

Calderon-Anyosa & Kaufman used interrupted time series to study the immediate impact of lockdown in Peru on non-Covid-19 deaths [41]. Moreover, Silva et al. introduced lockdown as a severe level of social distancing with higher enforcement [37]. They evaluated the effect of lockdown policy on the number of new Covid-19 cases and the number of new deaths in four cities in Brazil that implemented lockdown with different durations. For the interrupted time series analysis, Silva et al. considered only three variables, including time, trend, and level change after interventions. Their results showed the effect of lockdown on flattening the Covid-19 death curve [37].

Also, Thayer et al. studied lockdown policies and their effects on pandemics [38]. They considered the region population percentage, hospital beds, and annual air passenger arrivals to predict Covid-19 cumulative incidence. They used segmented regression to do the interrupted time series analysis as their data were over-dispersed. Thayer et al. also used mobility data as a covariate of segmented regression to show the robustness of their interrupted time series model. Because of the importance of mobility change, we also considered mobility change as one of the model predictors. We followed a similar method used by Thayer et al. to do interrupted time series analysis by segmented regression [38].

In this study, the prevalence of Covid-19 cases per million is considered the model’s outcome. The outcome depends on the daily cumulative percentage of vaccinated people, mobility changes, number of Covid-19 tests per population, intervention-related variables, and time-series specific variables (time and time squared). The variables for the interrupted time series model of interest are defined in Table 1.

**Table 1:** Model variables’ notations and definitions.

Dependent variable	
*PC*_*t*_	**P**revalence of new **C**ovid-19 **c**ases per million people at time *t*
**Independent variables**	
*t*	**T**ime starting from the beginning day of the third Covid-19 wave (*t* = 1 is March 7^th^ 2021) in Ontario
*CPV*_*t*_	**C**umulative **p**ercentage of Ontario **p**opulation who got the first dose of a Covid-19 **v**accine (Moderna, Pfizer-BioNTech, AstraZeneca/COVISHIELD, and Janssen) at time *t*
*MNA*_*t*_	**M**obility change in **n**on-residential **a**reas including retail and recreation, grocery and pharmacy, transit stations, and workplaces areas and parks at time *t* (showing how often people go out)
*MRA*_*t*_	**M**obility change in **r**esidential **a**reas at time *t* (showing people compliance and how often they stay at home)
*NT*_*t*_	**N**umber of Covid-19 **t**ests per population taken in Ontario at time *t*
*IL*_*t*_	**I**ntervention (stay-at-home order) **l**evel (severity) at time *t*
	${\;IL}_{t}\;=\;\left\{ \begin{array}{c} 0\;before\;intervention\;effective\;date\;(April\;19^{th}\;2021)\;\\ 1\;after\;intervention\;effective\;date\;(April\;19^{th}2021)\; \end{array}\right.$
*IT*_*t*_	**I**ntervention (stay-at-home order) **t**rend change at time t, which is the number of days since the intervention was implemented.
	${\;IT}_{t}\;=\;\left\{ \begin{array}{c} 0\;before\;intervention\;effective\;date\;\\ t-43\;after\;intervention\;effective\;date\; \end{array}\right.$
	43 is the number of days between the beginning day of the third Covid-19 wave (March 7^th^ 2021) in Ontario and the intervention effective day (April 19^th^, 2021)

The interrupted time series model is as follows. The model for the period before the intervention is:

$${PC}_{t}∼b_{0}+b_{1}t+b_{2}t^{2}+b_{3}{\;CPV}_{t}+b_{4}{{\;CPV}_{t}}^{2}+b_{5}{\;MNA}_{t}+{b_{6}MRA}_{t}+{b_{7}\;NT}_{t}+{\varepsilon^{\prime }}_{t}$$ (1)

The comprehensive model containing intervention variables for the period after the intervention is:

$${PC}_{t}∼b_{0}+b_{1}t+b_{2}t^{2}+b_{3}{CPV}_{t}+b_{4}{{CPV}_{t}}^{2}+b_{5}{MNA}_{t}+{b_{6}MRA}_{t}+{b_{7}NT}_{t}{+b}_{8}{IL}_{t}{+b}_{9}{IT}_{t}+\varepsilon_{t}$$ (2)

Where *b*_*i*_ are the regression coefficients and *ε*_*t*_ is the error term at time *t* and the effectiveness of the intervention is evaluated by *b*_8_ *IL*_*t*_ and *b*_9_ *IT*_*t*_ .

One point to be noted is the population was considered constant during the study period, so the effect of population density on Covid-19 prevalence (population density multiplied by a coefficient (*b*×population density)) was reflected as a constant number in the regression line’s intercept. As vaccination and testing are free in Ontario, no vaccine and testing costs were included in the model.

Table 2 states the correlation matrix of considered independent variables except for interrupted time-series specific variables, which are necessary components of ITS. Since the correlations are less than 0.8, there is essentially no multi-collinearity among these variables, and they can be considered independent. For example, Google defined non-residential mobility and residential mobility differently, and their correlation is less than 0.8, so they can both be considered in the model.

**Table 2:** Correlation matrix of covariates of ITS.

Correlations	Vaccination percentage	Mobility in non-residential areas	Mobility in residential areas	Test percentage
Vaccination percentage	1			
Mobility in non-residential areas	0.456	1		
Mobility in residential areas	-0.015	-0.729	1	
Test percentage	-0.342	-0.276	0.101	1

As the prevalence of Covid-19 cases over the period of interest (S1 Fig) had a non-linear relationship with a negative quadratic appearance, quadratic terms should be added to the model [42], like polynomial regression, which is an extension of linear regression with quadratic terms. Thus, time squared and cumulative vaccination percentage squared terms were added to the model to capture the apparent non-linearity.

According to highly cited research by Lauer et al. [43], the pre-Delta variant incubation period of Covid-19 for 97.5% of the people is 11.5 days with a 95% confidence interval between 8.2 to 15.6 days [43]. Since our time unit analysis is one day, 11.5 days were rounded up, so 12 days are considered the incubation period. Also, 12.5 days are the 95% percentile of incubation period distribution according to Li et al. [44]. Therefore, 12 days is a good selection with a proper confidence interval. This is the minimum time required to see the effect of the intervention on the outcome variable. The effective day of the stay-at-home order was determined to be April 19^th^, 2021 (April 7^th^ + 12 days). Based on the model mentioned above, the statistical hypothesis is:

$$H_{0}:\;b_{\left(i\right)}=0\;(\mathrm{or}\;p‐\mathrm{value}\;\;\mathrm{non}‐\mathrm{significant})\;i=0\leq 9$$ (3.1)

$$H_{1}:\;b_{\left(i\right)}\neq 0\&(p‐\mathrm{value}\;\mathrm{significant})\;i=0\leq 9$$ (3.2)

Two coefficients calculate the impact of the stay-at-home intervention on the Covid-19 prevalence (*b*_8_ and *b*_9_). Therefore, if these coefficients in the model were not zero and statistically significant, this means that the intervention was effective.

To implement interrupted time series analysis, NLME [45] and CAR [46] libraries of the R package were used. ITS preliminary analysis is done in S1 File to check for autoregressive residual and moving average. Results indicate that Autoregressive Residuals (AR) is 7, which means that residuals are dependent on their 7 previous values.

The Generalized Least Square (GLS) method, an extension of OLS, can consider both moving average and autoregressive errors [47] and was used to estimate model coefficients. The GLS estimates for the regression model is as follows:

$${\hat{\beta }}_{GLS}\;=\;{\left(X^{T}\Omega^{-1}X\right)}^{-1}X^{T}\Omega^{-1}y$$ (4)

where Ω is the covariance matrix of residuals because they are dependent.

## Results

The estimates and Confidence Intervals (CIs) from GLS analysis are reported in Table 3. All significance tests were two-sided. Compared to the OLS Standard Error (SE) in ITS preliminary analysis in S1 File, the SE of the GLS model with AR (7) is less, showing that GLS is a better fit.

**Table 3:** GLS results of R software with AR (7).

Coefficient	Related variable of the coefficient	GLS+AR (7)			
		Coefficients estimates	95% CI of estimates	*t-*value	*p*-value
*b*_0_	Intercept	0.496	[-41.01, 42.00]	0.0238	0.98
*b*_1_	Time (*t*)	-3.655	[-6.01, -1.30]	-3.0966	0.003*
*b*_2_	Time^2^	0.101	[0.02, 0.18]	2.5048	0.014*
*b*_3_	Vaccination percentage (*CPV*_*t*_)	1668.99	[755.01, 2582.97]	3.6370	≤0.001***
*b*_4_	Vaccination percentage^2^	-2260.60	[-3101.1, -1420.03]	-5.3563	≤0.001***
*b*_5_	Mobility in non-residential areas (*MNA*_*t*_)	-0.159	[-0.65, 0.33]	-0.6415	0.52
*b*_6_	Mobility in residential areas (*MRA*_*t*_)	-1.801	[-3.10, -0.43]	-2.6180	0.011*
*b*_7_	Number of tests (*NT*_*t*_)	14403.47	[8629.2, 20177.64]	4.9682	≤0.001***
*b*_8_	Intervention level change (*IL*_*t*_)	-32.03	[-47.34, -16.72]	-4.1675	≤0.001***
*b*_9_	Intervention trend change (*IT*_*t*_)	-13.19	[-14.97, -11.4]	-14.7135	≤0.001***
Residual standard error			17.07 (Degree of freedom = 76)		

*** at 0.001 level; ** at 0.01 level

* at 0.05 level.

According to the GLS results, the stay-at-home order caused significant changes in the level of the Covid-19 case prevalence (-32.03) and its trend (-13.19) with *p-*values ≤ 0.001. While the vaccination percentages of people had significant non-zero coefficients and helped control Covid-19 spread, the results showed that the non-pharmaceutical interventions increased the overall effectiveness and avoided a Covid-19 surge. As a result, the stay-at-home order significantly decreased the trend of Covid-19 spread over the period studied by about 14 persons per million per day. It caused a reduction of about 33 Covid-19 cases per million as an immediate effect of the intervention. Also, the number of tests taken per day was a significant predictor of Covid-19 prevalence (*p*-value ≤ 0.001) in the GLS model. It is possible that correlations between non-residential area mobility change and the stay-at-home order intervention caused the non-residential area mobility coefficient to be non-significant (*p*-value = 0.52). The Pearson correlation of non-residential area mobility change with intervention level change and intervention trend change is 0.26 (*p*-value ≤ 0.05) and 0.54 (*p*-value ≤ 0.01), respectively. SPSS 27 software was used to derive the bivariate correlation test results.

**Fig 6:**
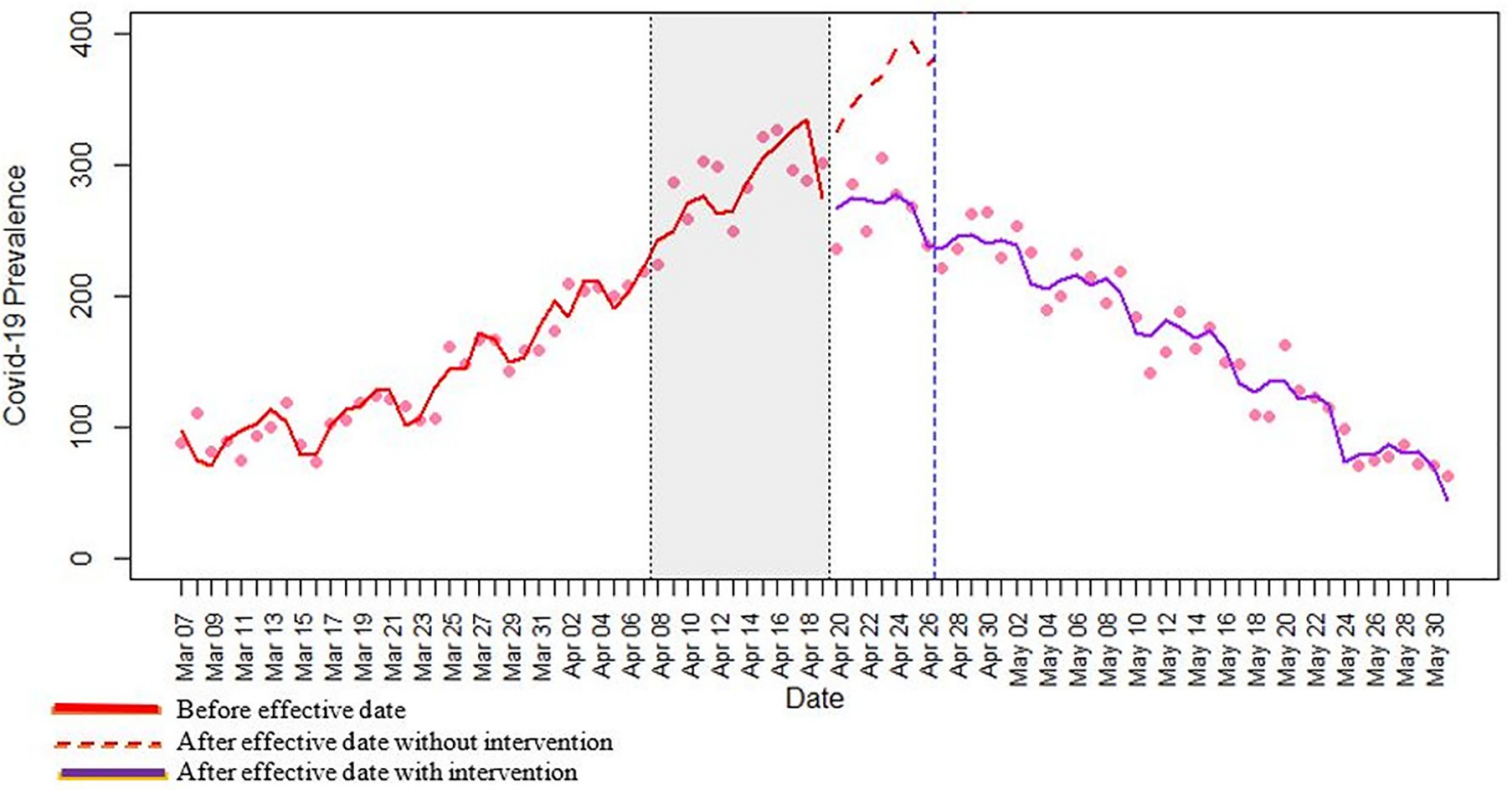
Covid -19 prevalence pattern with and without a stay-at-home order.

Fig 6 shows the Covid-19 prevalence versus time. Although other variables are in the model, these two variables have been chosen to simplify the illustration. The red line shows the time series regression line. The gray box shows the time (12 days) that took governmental restrictions to show their effect (Covid-19 incubation period). The red dashed line projects what would have happened if there were no restrictions for a short period of a week represented by the blue dashed line. Clearly, vaccination was not enough by itself, and there would have been a Covid-19 surge if strict restrictions had not been employed simultaneously.

## Discussion

To see what would have happened if Ontario had imposed no stay-at-home order, the coefficients related to the intervention were ignored, and a counterfactual pattern was obtained by the simplified model (${\;PC}_{t}∼b_{0}+b_{1}t+b_{2}t^{2}+b_{3}{\;CPV}_{t}+b_{4}{{\;CPV}_{t}}^{2}+b_{5}{\;MNA}_{t}+{b_{6}MRA}_{t}+{b_{7}\;NT}_{t}+{\varepsilon^{\prime }}_{t}$) (dashed red line of Fig 6). The effect of the stay-at-home intervention was calculated by comparing counterfactual estimates, which is the baseline of analysis with the actual number of Covid-19 reported cases. The prevalence of Covid-19 cases a week after the intervention effective date of April 19^th^ is compared to its counterfactual estimate on April 26^th^, 2021. The Covid-19 prevalence per million was about 239 on April 26^th^, which would have been approximately 377 if there was no stay-at-home order. So, this preventive measurement caused a 36.6% reduction in the prevalence of Covid-19 cases a week after the intervention effective date (April 26^th^, 2021). It is aligned with the Fowler et al. study that showed a stay-at-home order is associated with about a 30.2% reduction in Covid-19 incidence after a week [15]. However, their study was related to the first wave, and our results confirm that a stay-at-home order was still effective during the third wave.

Due to the time coefficient, the prevalence of Covid-19 cases had a decreasing trend (-3.655 persons per million/day), possibly the combined result of the continuing increase in vaccination and the nature of epidemy. Still, the intervention accelerated this reduction (-13.18 persons per million/day). These strict interventions led to a sharp drop rather than a sustainable peak, thus reducing infections and saving lives. Hence, the other countries that started vaccination late can benchmark from Ontario.

Applying polynomial regression found that cumulative percentage of first dose vaccinated people and its quadratic form significantly affected Covid-19 incidence. Covid-19 prevalence decreased by 2260.603 multiplied by the first vaccination percentage squared. When the cumulative vaccination percentage reached 74%, the cumulative first dose vaccinated percentage and its quadratic form caused a reduction in the prevalence of Covid-19 cases. These results align with the Shen et al. study, showing that 50% to 80% coverage is required for a “moderate” vaccine to be effective [48]. Since the effectiveness of the first dose vaccine is less than two vaccine doses, it can be considered a moderate one.

When people spend more time in their homes, the mobility in residential areas increases. Ontario’s residential areas’ mobility increases showed Ontarian adherence to the stay-at-home order that caused a decrease (-1.801) in the number of people per million who got Covid-19 during the third wave. As a result, policymakers in all countries are encouraged to implement policies like a stay-at-home order limiting non-residential mobility and increasing residential mobility. Furthermore, our results concurred with Thayer et al. [38], which showed non-residential mobility decreased after the new restrictive policy announcement. However, we found that non-residential area mobility changes did not have a significant relationship with Covid-19 prevalence, probably because people got frustrated with the stay-at-home order in May and spent more time out than the baseline (shown in Fig 4). So, non-residential mobility may not be a good measurement of compliance with a stay-at-home order.

The prevalence of Covid-19 cases is related to the number of Covid-19 tests taken [49]. This is also shown by the significant positive coefficient of the number of tests in our results. The reduction in the Covid-19 incidence rate could also have resulted from the reduction in the number of Covid-19 tests in May, shown in Fig 1. A mass testing policy [49], which was not implemented in Ontario, might have helped to reduce Covid-19 cases by additional testing and isolating positive cases from the general public [50]. Also, Lyng et al. showed that increasing test frequency would reduce Covid-19 prevalence after a period [51]. As shown in Fig 1, another concern is the decrease in the number of tests on the weekends. Policymakers must provide a solution for this because it results in unawareness of positive cases that can infect more people.

### Limitations

One of the limitations of this study is that only clinically diagnosed Covid-19 cases were counted. At the same time, there might be positive Covid-19 cases not diagnosed if patients avoided tests or did not have severe symptoms. Even so, this issue covers the entire studied period, not just the period after the stay-at-home order. Therefore, undiagnosed cases would not significantly affect the intervention results. However, Z-number fuzzy numbers, which combine the expert’s subjective opinion with real data, are suggested to model this uncertainty for future research [52]. Moreover, Google stated that the mobility reports are based on Google account users who opt-in for location history sharing, and this might not represent the general behavior of society during the period covered by this study (Google [29]).

## Conclusion

This paper used interrupted time series to analyze the effectiveness of several policies accumulated in a “stay-at-home” order, not one single policy. Quadratic terms have been added to capture the non-linearity in the Covid-19 prevalence model. Several contributions distinguish this study from previous papers. For instance, this study considered the vaccination policy and vaccinated people, which affected the dynamic of the Covid-19 epidemy. This study investigated NPIs during the third wave of Covid-19 and its interactions with pharmaceutical interventions (vaccinations), while most of the previous papers only studied NPIs during the first wave. Also, this study considers the compliance of people with restrictive policies using Google mobility reports on the Ontario community.

Despite the opinions of small business owners that were not satisfied with the second stay-at-home order, ITS analysis results show the importance of the stay-at-home order in controlling Covid-19 prevalence during the third wave. This research should help to increase public awareness of the need for strict preventive measures even while more people get vaccinated. The stay-at-home order caused a significant reduction in both level (-472 person/day) and trend (-195 person/day) of new Covid-19 cases. Indeed, the shutdown and the stay-at-home order of the Government of Ontario in early April, one month after the third wave started in early March, caused a 37% reduction in Covid-19 prevalence after the intervention in late April. Also, our results show a high correlation between the number of tests and Covid-19 cases. Currently, only symptomatic people are tested in Ontario, but to eliminate Covid-19 cases, mass testing is required. Researchers can determine the adequate percentage of the population that needs to be tested to implement a mass testing policy in future studies. Moreover, the effect of different Covid-19 control policies on the number of deaths and hospitalizations should be taken into account in the future considering the high health care costs.

## Supporting information

S1 Fig Daily number of new Covid-19 cases in Ontario since the pandemic beginning till 10^th^ June 2021. (TIF)

S1 File Interrupted Time Series (ITS) preliminary analysis. Checking autoregressive residual and moving average.
(DOCX)
