# Supplementary figures and images for: The effect of the Ontario stay-at-home order on Covid-19 third wave infections including vaccination considerations: An interrupted time series analysis
Source: PLoS One. 2022 Apr 6;17(4):e0265549. doi: 10.1371/journal.pone.0265549 (PMC8986007; doi:10.1371/journal.pone.0265549)

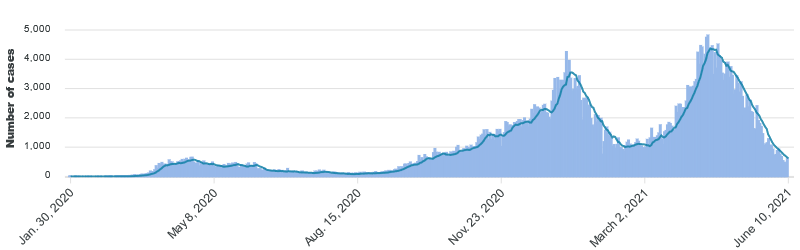

Supplement: S1 Fig — (TIF) [file pone.0265549.s001.tif]
