# Supplementary material for: The effect of the Ontario stay-at-home order on Covid-19 third wave infections including vaccination considerations: An interrupted time series analysis
Source: PLoS One. 2022 Apr 6;17(4):e0265549. doi: 10.1371/journal.pone.0265549 (PMC8986007; doi:10.1371/journal.pone.0265549)
Supplement: S1 File — Checking autoregressive residual and moving average. (DOCX) [file pone.0265549.s002.docx]

**Supplementary material A**

**ITS Preliminary Analysis**

To do ITS preliminary analysis, the Ordinary Least Squares (OLS) method is used to estimate regression model parameters by equation (A.1) that minimizes the sum of squared errors. OLS is one of the most common estimation procedures, which assumes that the error terms are independent [1]. So, we need to check the OLS results for potential autocorrelation.

$\hat{\beta}_{OLS}=\left( X^{T}X \right)^{-1}X^{T}y$ (A.1)

Where $\hat{\beta}$ is a vector of OLS estimation for the regression coefficients ($b_{0}, \ldots, b_{9}$), $X$ is a vector of predictors and $y$ is the response variable (${PC}$ in this study). The OLS results are presented in Table A.1. Multiple R squared is 0.9504, which means that the results predict 95.04 % of the variance in the dependent variable. Also, the *p*-value of the F statistic is less than ${2.2\times10}^{-16}$ which is significant and means that the null hypothesis is rejected, and the selected independent variables successfully explain the dependent variable.

Table A.1. OLS results of R software

| **Coefficient** | **Related variable of Coefficient** |  | **OLS** | |
| --- | --- | --- | --- | --- |
|  |  | **Coefficients**  **estimates** | ***t-*value** | ***p*-value** |
| $b_{0}$ | Intercept | -22.6 | -0.834 | 0.40 |
| $b_{1}$ | Time ($t$) | -3.767 | -2.210 | 0.03* |
| $b_{2}$ | Time^2^ | 0.0625 | 1.120 | 0.27 |
| $b_{3}$ | Vaccination percentage (${CPV}_{t}$) | 1897 | 3.059 | 0.003 ** |
| $b_{4}$ | Vaccination percentage^2^ | -1776 | -2.820 | 0.006 ** |
| $b_{5}$ | Mobility in non-residential areas (${MNA}_{t}$) | -0.0866 | -0.295 | 0.77 |
| $b_{6}$ | Mobility in residential areas  (${MRA}_{t}$) | -0.586 | -0.801 | 0.43 |
| $b_{7}$ | Number of tests (${NT}_{t}$) | 13730 | 4.376 | ≤0.001 *** |
| $b_{8}$ | Intervention level change (${IL}_{t}$) | -28.33 | -2.599 | 0.01 * |
| $b_{9}$ | Intervention trend change (${IT}_{t}$) | -12.95 | -9.719 | 0.000 *** |
| Residual standard error | | 17.6 (Degree of freedom 76) | | |
| *** at 0.001 level; ** at 0.01 level; * at 0.05 level | | | | |

OLS assumes that residuals are identical and independent (iid). In the preliminary analysis, this hypothesis was checked. There are different methods to check whether the residuals of the OLS model are correlated Saki et al., (2021) [2]. Figure A.1 illustrates residuals versus time.


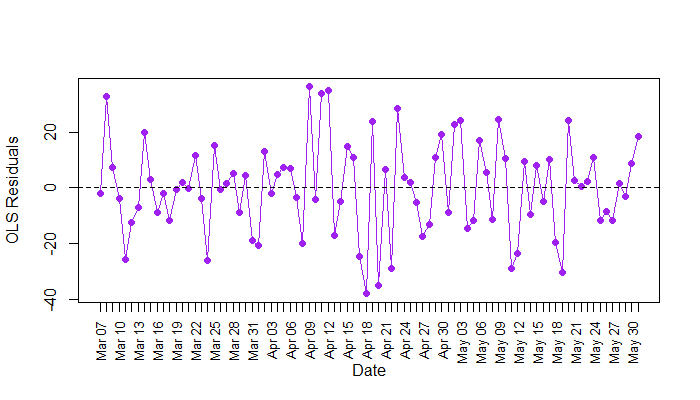


**Figure A.1.** OLS residual plot

To check for autocorrelation, we used an Auto Correlation Function (ACF) plot, which plots the correlation of a variable with itself over time, and Partial ACF (PACF) plot, which plots the correlation of a variable with itself over time after removing the linear component [3].

Figure A.2 shows the ACF and PACF plots of the OLS model residuals with a maximum of 20-day lags to see how far the autocorrelation (if any) extends. Since ACF shows exponential decay (pink dashed line), autoregressive (AR) residuals should be considered. Autoregressive errors mean that the error term has some relationship with the error term in previous periods plus a random error ($\varepsilon_{t}=\varphi_{1}\varepsilon_{t-1}+\nu_{t}$ for AR=1) [4]. However, since PACF does not show a decay, considering a moving average between residuals is not needed. Instead, the spikes in PACF determine the order of autoregressive series that should be considered. Spikes shown by red lines happen when the value of ACF/PACF exceeds the two thresholds (blue dashed lines) of the 95% confidence band. There are two spikes on days 7 and 16 of the PACF graph. As the spike in lag 7 is the largest, AR (7) should be considered for the model, meaning that the residuals are dependent on their 7 previous values.


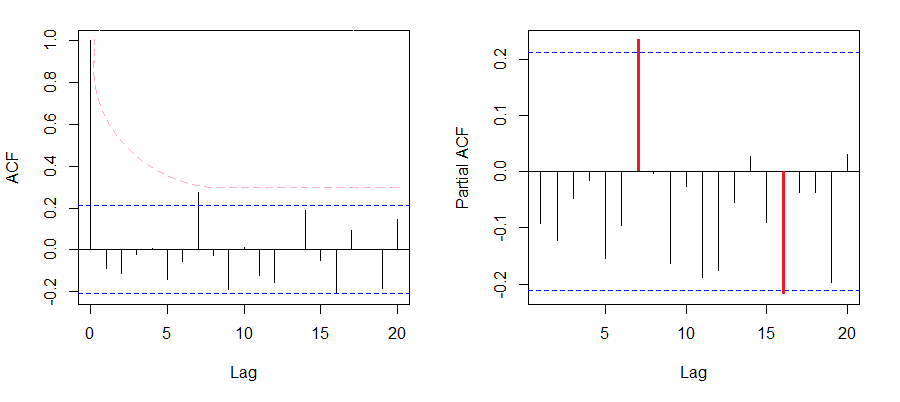


Figure A.2. ACF and PACF plot of OLS model residuals

The Durbin-Watson test to check autoregressive residuals confirms AR (7). This test provides statistical values between 0 and 4 [5]. The value 2 indicates no correlation among errors, values over 2 indicate a positive correlation, and values under 2 indicate a negative correlation [5]. The Durbin-Watson test was employed for lags of 1 to 20 inclusive. The calculated value of this statistic is 1.332 (*p*-value<0.01) and 2.175 (p-value<0.05) for 7- and 16-day lags, respectively. The 7 days lag, which has a more significant statistic, was chosen. The results of the Durbin-Watson test are reported in Table A.2.

Table A.2. Durbin-Watson test results

| **Lag (days)** | **statistics** | ***p*-value** | **Lag (days)** | **statistics** | ***p*-value** |
| --- | --- | --- | --- | --- | --- |
| 1 | 2.1686 | 0.97 | 11 | 2.1000 | 0.15 |
| 2 | 2.1635 | 0.74 | 12 | 2.1412 | 0.06 |
| 3 | 1.9821 | 0.82 | 13 | 1.7869 | 0.84 |
| 4 | 1.9213 | 0.69 | 14 | 1.3937 | 0.10 |
| 5 | 2.1807 | 0.34 | 15 | 1.8641 | 0.41 |
| 6 | 2.0026 | 0.92 | 16 | 2.1750 | **0.02** |
| 7 | 1.3322 | **0.002** | 17 | 1.5706 | 0.91 |
| 8 | 1.9136 | 0.97 | 18 | 1.7148 | 0.44 |
| 9 | 2.2343 | 0.08 | 19 | 2.0764 | 0.01 |
| 10 | 1.8343 | 0.88 | 20 | 1.3962 | 0.49 |

**References**

1. Ayinde K, Lukman AF, Rauf RI, Alabi OO, Okon CE, Ayinde OE. Modeling Nigerian Covid-19 cases: A comparative analysis of models and estimators. Chaos, Solitons and Fractals. 2020;138: 1–16. doi:10.1016/j.chaos.2020.109911

2. Saki M, Ghanbari MK, Behzadifar M, Imani-Nasab MH, Behzadifar M, Azari S, et al. The impact of the social distancing policy on COVID-19 incidence cases and deaths in Iran from february 2020 to january 2021: Insights from an interrupted time series analysis. Yale J Biol Med. 2021;94: 13–21.

3. Bernal JL, Cummins S, Gasparrini A. Interrupted time series regression for the evaluation of public health interventions: A tutorial. Int J Epidemiol. 2017;46: 348–355. doi:10.1093/ije/dyw098

4. ArunKumar KE, Kalaga D V, Sai Kumar CM, Chilkoor G, Kawaji M, Brenza TM. Forecasting the dynamics of cumulative COVID-19 cases (confirmed, recovered and deaths) for top-16 countries using statistical machine learning models: Auto-Regressive Integrated Moving Average (ARIMA) and Seasonal Auto-Regressive Integrated Moving Averag. Appl Soft Comput. 2021;103: 107161. doi:10.1016/j.asoc.2021.107161

5. Fox J, Weisberg S. Time-series regression and generalized least squares: an appendix. An R Companion to Appl Regres. 2018; 1–8. Available: http://tinyurl.com/carbook.
